# News and Events of the Week

**Published:** 1910-04-09

**Authors:** 


### News and Events of the Week.

The Radium Institute will be opened next autumn, probably in the month of October.

Of the £30,000 required for the Building Fund of the Royal Society of Medicine only £7,000 odd has so far been raised.

The annual general meeting of the Medical Defence Union will be held at the Royal Station Hotel, Hull, on Thursday, May 19, at 5 p.m., when the annual report and audited balance-sheet of accounts will be presented, and the statutory resolutions brought forward.

A Departmental Committee has been appointed by the Postmaster-General (the Right Hon. Herbert Samuel, M.P.) to inquire into the disease known as "telegraphists' cramp," and to report what means may be adopted for its prevention. Sir John N. Barran, M.P., will act as chairman of the committee, the other members of which will include Dr. Harold Theodore Thompson, assistant physician to the London Hospital, and Dr. J. Sinclair, second medical officer of the Post Office.

The second annual series of demonstrations illustrating specimens contained in the museum of the Royal College of Surgeons will be delivered in the theatre of the Royal College of Surgeons by the conservator, Professor Arthur Keith, and by the pathological curator, Professor S. G. Shattock. Professor Keith will lecture on April 15 and 22 on "Misplacements, Malformations, and Cysts"; and Professor Shattock will lecture on April 18 and 25 and May 2 on "Cancer, Tuberculosis, and Specimens Illustrating Repair."

The Chester Historical Pageant, which opens on Monday, July 18 next, and continues until July 23, is one of the most interesting of these historical exhibitions which are nowadays so deservedly popular. We have received the Pageant Book, which is an attractively got-up pamphlet, well illustrated, giving all details about the performances, and also valuable information regarding Chester and its environs. The pageant is under the distinguished patronage of T.R.H. the Prince and Princess of Wales. Special railway arrangements have been made, and a Housing Committee has been formed whose sole function is to arrange for the comfort of visitors to the pageant. Applications for apartments should be made, as soon as possible, to the Secretary, Housing Committee, Pageant House, Chester.

On the first of April two important Acts came into operation. The first is the Midwives Act, which provides that no uncertificated midwife may attend a confinement case except in an emergency, and then only under medical supervision. The second is the Asylum Officers' Superannuation Act. The asylums authorities have been busy classifying the different officials in accordance with the provisions of the Act, and it appears that the great majority of the officers in asylums have as part of their ordinary duty the care of the insane, and therefore come under the more beneficial scale of pensions provided by the Act. The Home Secretary has stated in reply to a question by Sir William Collins that he does not consider that he has any power under the Act to overrule the classifications, and will not consequently undertake to be a court of appeal in regard to them.

Mr. Walter Runciman, President of the Board of Education, has appointed Mr. F. G. Ogilvie, C.B., to a new post of Secretary of the Board for the Science Museum, Geological Museum and Geological Survey. Mr. E. K. Chambers has been appointed to succeed him as Principal Assistant-Secretary of the Technological Branch of the Board.

A Special Meeting of the Medical Society of London will be held on Monday, April 18, at 8.30 p.m., at which a paper on "Inheritance in Hæmophilia" will be read by Dr. W. Bullock and Dr. Paul Fildes. The following are expected to be present and take part in the discussion: Sir Almroth Wright, M.D., Professor W. Osler, Mr. E. Nettleship, Dr. Parkes Weber, Dr. H. D. Rolleston, Professor Karl Pearson, Professor W. Bateson, Dr. Gossage and others.

The provincial sessional meeting of the Royal Sanitary Institute takes place at Plymouth to-day. Yesterday's meeting was held in the Western Law Courts, Guildhall, Plymouth, at 7.30 p.m., when a discussion took place on "The Removal of Town Filth," opened by Mr. J. Paton, and on "The Relative Value of Disinfectants in the Control of Diseases," opened by Dr. O. Hall, D.P.H., M.O.H., Devonport. The chair was taken at 7.30 p.m. by Colonel J. Lane Notter, R.A.M.C., M.A., M.D. To-day's programme is as follows: Meet at "The Octagon," Union Street, and proceed to visit Messrs. James & Rosewall's lead works, at 10 a.m. Trams from Derry's Clock to "The Octagon," fare 1d. 12.15 p.m.—Luncheon at the Globe Restaurant. No luncheon tickets will be issued, but a charge of 2*s*. 6*d*. each person will be made at the tables. 2.30 p.m.—Reassemble at Millbay Station, Great Western Railway, and proceed to visit the Plymouth Burrator Water Works at 2.45 p.m. Return fare 1s. 3d.

Mr. Walter Runciman, President of the Board of Education, has appointed a Departmental Committee to consider and report upon various questions in regard to the present condition and the future development of the valuable collections comprised in the Board's Science Museum at South Kensington and Geological Museum in Jermyn Street. In particular the committee is asked to advise him (*a*) as to the precise educational and other purposes which the collections can best serve in the national interests; (*b*) as to the lines on which the collections should be arranged and developed, and possibly modified, so as more effectively to fulfil these purposes; and (*c*) as to the special characteristics which should be possessed by the new buildings which it is hoped will shortly be erected on the South Kensington site to house these collections, so as to enable the latter to be classified and exhibited in the manner most fitted to accomplish the purposes they are intended to fulfil. The committee is as follows: Sir Hugh Bell, Bart., D.C.L. (chairman); J. J. Dobbie, F.R.S., LL.D.; Sir Archibald Geikie, K.C.B., F.R.S.; R. T. Glazebrook, D.Sc., F.R.S.,; Andrew Laing, M.I.C.E.; the Hon. Sir Schomberg McDonnell, K.C.B., C.V.O.; Sir William Ramsay, K.C.B., F.R.S., LL.D.; Professor W. Ripper, D.Eng., M.I.C.E., Hon.M.I.Min.E.; Sir W. H. White, K.C.B., F.R.S., LL.D., with Mr. F. G. Ogilvie, C.B., one of the Secretaries of the Board of Education as secretary.

Surgeon-Major Thomas Egerton Hale, V.C., C.B., a Crimean veteran, who died on December 25, aged seventy-seven, left estate valued at £10,518 gross, with net personalty £7,175.

The arrangements for the Festival of Empire and the Pageant of London, which will be held at the Crystal Palace early in May, are nearing completion. The profits of the festival and pageant will be devoted to the King Edward VII. Hospital Fund.

Mr. John William Taylor, F.R.C.S., of Newhall Street, Birmingham, and of the Red House, Northfield, Worcester, the late Professor of Gynæcology in the University of Birmingham, who died on February 26, in his sixtieth year, has left estate valued at £25,294 gross and £23,457 net.

Dr. C. R. Huxley, Principal of the Kent House Nursing Home, Beckenham, who was charged at the Bromley Police Court this week with unlawfully wounding a schoolboy by shooting him in the right eye with a gun, has been remanded, being bound over in the sum of £200 and ordered to find two sureties of £100 each.

The following degrees have been conferred *honoris causa* by the University of Aberdeen :—LL.D. : Dr. James Mackenzie, M.D. (Edin.), London; Mr. James Niven, M.A. (Aberd.), M.B. (Cantab.), Medical Officer of Health, Manchester; and Dr. Alexander Ogston, C.M., M.D. (Aberd.), Emeritus Professor of Surgery, Aberdeen.

"The Social Calendar for 1910" is an attractively got-up little volume with a list of the coming social events of the year. Besides telling one when the horse show begins, for example, it contains hints, drawn from authoritative sources, on the niceties of Court dress, and, say, the origin and arrangements of Henley Regatta.

A Council meeting of the Association of Medical Officers of Health was held on March 24 at the Holborn Restaurant, with Dr. Crookshank in the chair. There was a good atendance. Dr. Belilios, the hon. secretary, reported the accession of new members and read letters offering co-operation from societies and persons of influence. A cordial vote of thanks was passed to the Poor Law Medical Officers' Association for their resolution, and appreciation was expressed at the proposed action of other bodies. It was reported by the Chairman and Dr. Gough that the Richmond and Altrincham divisions of the British Medical Association had passed resolutions instructing their delegates at the next representative meeting to press for the rescindment of minute 234, and it was noted that other divisions were considering the matter. The further steps which had been taken were reported and other measures agreed on. A full report of the meeting inaugurating the Association is to be circulated among interested persons and bodies. It was agreed to hold a general meeting in London during the annual meeting of the British Medical Association and to communicate with the Council of the latter body on the subject. It was further resolved that the meeting should be followed by a dinner, to which influential guests would be invited. The Hon. Secretary reported that at the last meeting of the Home Counties branch of the Society of Medical Officers of Health, Dr. Crookshank (Chairman of the Association) had been nominated for the presidency of the Society in succession to Dr. Cooper Pattin. It was unanimously agreed that steps be at once taken to support Dr. Crookshank at the election, which takes place at 1 Upper Montague Street, Russell Square, on April 8, at 5 p.m.

